# Attentional bias toward food in binge eating disorder: baseline differences and the limits of attention modification training

**DOI:** 10.1186/s40337-025-01450-4

**Published:** 2025-11-05

**Authors:** Lynn Sablottny, Dustin Werle, Jennifer Svaldi, Nicole Thörel, Detlef Caffier, Brunna Tuschen-Caffier

**Affiliations:** 1 Department of Clinical Psychology and Psychotherapy, University of Freiburg, Engelbergerstraße 41, 79085 Freiburg, Germany; 2 Department of Clinical Psychology and Psychotherapy, University of Tuebingen, Schleichstraße 4, 72074 Tuebingen, Germany

**Keywords:** Attentional bias, Attentional bias modification, Binge eating disorder, Dot-probe paradigm, Eye-tracking, Food

## Abstract

**Background:**

Attentional processes toward high-calorie foods contribute to the maintenance of binge eating disorder (BED) and have been targeted by attentional modification trainings (AMTs). In this study, we quantified food-related attentional bias (AB) in individuals with BED versus control groups with normal-weight (NCG) and overweight (OCG), and evaluated whether AMT effects would persist after one week and generalize to novel stimuli.

**Methods:**

We assessed eating pathology and AB in 135 participants (BED: *n* = 72; NCG: *n* = 32; OCG: *n* = 31). We used a dot-probe paradigm with concurrent eye-tracking and reaction-time measures. Sixty-one participants with BED were then randomized to four sessions of AMT or placebo training. All participants with BED underwent re-evaluations of AB and eating pathology one week after the final training session.

**Results:**

At baseline, the group with BED presented significantly greater AB toward high-calorie food cues than both the NCG and OCG did. One week post-training, no differential effects of AMT were observed: both the AMT and placebo groups showed modest, nonspecific reductions in initial fixation duration bias and reaction-time variability. Correlations between changes in AB toward food and eating pathology were small and not significant.

**Conclusions:**

The presence of a food-related AB in individuals with BED was confirmed. However, AMT did not yield sustained or generalized modifications in attentional processing beyond those observed in the placebo condition. Nonspecific improvements may reflect enhanced overall attentional control or general exposure effects. Future research should isolate the active components of AMT and explore strategies to increase its ecological validity.

*Trial registration:* Registered in the German Clinical Trials Register (DRKS00012984) on 2017-11-30.

**Supplementary Information:**

The online version contains supplementary material available at 10.1186/s40337-025-01450-4.

## Abstract (plain language summary)

People with binge eating disorder (BED) tend to pay more attention to high-calorie foods automatically. To study this, we showed images of food and neutral items side by side while tracking eye movements and reaction times. As expected, individuals with BED looked longer at food pictures than people with normal-weight and overweight without BED did. Next, we tested whether a brief computer-based training (attentional modification training, or AMT) could sustainably reduce this food-focused attention. Sixty-one participants with BED were randomly assigned to either four real AMT sessions or sham training that did not target attention. One week later, we repeated the attention tests using new food images. Both groups showed small, similar improvements in how quickly they shifted attention away from food; no lasting or unique benefit of the AMT could be found. These minor attentional changes were not strongly linked to reductions in binge eating episodes or cravings. It appears that simple repeated exposure to food images or general practice effects, rather than the specific AMT, may explain the slight improvements. In summary, people with BED do have a stronger focus on high-calorie foods, but our short AMT did not produce lasting changes in attention or clearly improved their eating behaviors. Future research should identify which training elements truly help and how to make effects last in everyday life.

**Supplementary Information:**

The online version contains supplementary material available at 10.1186/s40337-025-01450-4.

## Background

Binge eating disorder (BED) is characterized by recurrent binge eating episodes, during which individuals consume a large amount of food within a discrete period of time and experience a loss of control over their eating behavior. Feelings of shame, disgust and/or guilt often accompany these episodes and lead to significant distress [1]. While cognitive-behavioral psychotherapy is effective in reducing BED symptoms [2–4], binge eating persists in 30–50% of patients with BED after successful therapy [5–7]. Thus, further research is needed to identify the factors perpetuating binge eating to inform the development of alternative interventions or improvements to existing treatments [8].

One relevant mechanism in binge eating is attentional processes toward affective stimuli, such as food cues (e.g., 9–11). Several studies have shown that individuals with BED display an attentional bias (AB) toward food stimuli (for a review, see [11]). This is evident in increased and faster attentional focus, longer dwell times, and difficulties in disengaging from food stimuli [12–20]. Electrophysiological results also point to an AB toward food in individuals with BED [21, 22], including results from the sample presented here [23].

This selective perception of food stimuli can be explained by incentive-sensitization theory (IST), which was originally developed to understand drug addictions [24, 25]. The IST proposes that repeated exposure to rewarding stimuli—such as palatable food—induces neuroadaptive changes in the dopaminergic system, leading to increased attentional capture and craving responses [26, 27].

However, the resulting AB toward food stimuli is likely not a stable phenomenon uniformly pronounced across different contexts or time points [28]. While the high incentive value of food draws attention to these stimuli, competing motivational factors may simultaneously influence attentional allocation [29]. For example, food cues may also evoke concerns about unwanted weight gain or negative emotions associated with binge eating [29, 30]. Such motivational conflict can manifest as an approach-avoidance pattern in attention, arising when consumption is associated with immediate positive reinforcement but long-term negative consequences [31, 32]. This variability in attentional engagement is not adequately captured by mean AB scores [33], potentially explaining the heterogeneous findings for AB toward food across different groups [11, 30, 34]. Dynamic AB measures that capture this variability have shown promise in explaining additional variance in various disorders [35, 36]. However, no studies to date have examined whether individuals with BED differ from healthy controls in terms of such dynamic measures.

Investigating attentional processes in BED is relevant, because, from a theoretical perspective, they may causally contribute to food intake and eating disorder symptomology [37], even though experimental evidence is scarce. To address this research gap, several studies have employed attentional modification training (AMT) paradigms to experimentally alter AB toward food across various populations [37–50]. AMTs have demonstrated promising effects in reducing AB [51] and alleviating symptoms in various disorders, including anxiety disorders [52] and substance use disorders [53]. However, in samples with binge eating, it remains unproven whether AMT-induced reductions in AB actually lead to clinically meaningful decreases in food intake or eating-disorder symptoms [46]. For example, in our recent randomized trial with individuals with BED, four sessions of active AMT produced a significantly greater reduction in AB toward food than did placebo training. Nevertheless, this change in attentional bias did not translate into greater decreases in food consumption or binge eating frequency in the AMT group than in the placebo group [50].

Building on these unexpected findings, the present study aims to further clarify and extend our understanding by analyzing additional data from the same trial. For AMT to produce meaningful benefits, its effects must generalize beyond the specific stimuli used in training—extending to untrained food cues and across varied contexts [54]. Moreover, participants must exhibit a pronounced baseline AB toward food that is demonstrably linked to their eating-disorder symptoms, since without this, even substantial lab-based bias reductions are unlikely to translate into real-world improvements [54, 55].

Therefore, our objectives are twofold: First, we will quantify the magnitude of the food-related AB in the group with BED relative to control groups with overweight (OCG) and normal weight (NCG), hypothesizing that the group with BED would exhibit a greater bias toward food. We predict that greater bias toward food will correlate with more severe eating-disorder pathology, especially within the group with BED. Second, we will assess whether reductions in food-related AB achieved during AMT transfer to an untrained context. To this end, we measured AB before and one week after training using a novel stimulus set, hypothesizing that the AMT group would exhibit significant bias reductions, wheras the placebo group would show minimal or no change.

In addition to these preregistered hypotheses, we will explore whether changes in AB toward food correlate with changes in subjective cravings and binge eating frequency. Additionally, we will examine baseline differences and changes in dynamic AB measures (dwell time bias variability and reaction time [RT] bias variability).

## Methods

The study presented here is part of a DFG-funded two-center study carried out at the Universities of Freiburg and Tübingen (TU 78/10 − 1; SV 83/5 − 1). The project has been preregistered at the German Clinical Trials Register (ID: DRKS00012984; https://drks.de/search/en/trial/DRKS00012984). Owing to the complex study design, not all data and results from this project can be presented in one publication. Self-report measures pertaining to the sample are therefore also reported in other publications (23, 50). However, all the eye-tracking data presented are original.

### Samples

For the group with BED, the inclusion criteria were a BED diagnosis according to the Diagnostic and Statistical Manual of Mental Disorders (DSM-5; [1]), age 18–69 years and BMI 17.5–45. The control groups included participants aged 18–69 with no lifetime history of an eating disorder. For inclusion in the NCG, a BMI of 17.5–25 was required, whereas the OCG was matched to the group with BED by BMI. Across all groups, efforts were made to achieve comparable distributions of age and gender. The exclusion criteria for all groups were a vegan diet, high suicide risk, comorbid psychotic or bipolar illness, alcohol/substance dependence, serious medical complications, pregnancy or lactation. To ensure that improvements in AB for food and BED symptoms could be attributed to AMT, further exclusion criteria were ongoing psychotherapy and participation in weight loss programs.

Eating disorders were assessed via the Eating Disorder Examination (EDE; [56, 57]). All other diagnoses were made via the Structured Clinical Interview (SCID I + II) for DSM-IV [58]. All the participants provided informed consent prior to study participation. The participants with BED received 132€, and the control participants received 41€, due to fewer sessions. Recruitment occurred via flyers, social media, and ads.

Simulated power analyses using the effect sizes reported by Schmitz and Svaldi (2017) [37] indicated that in a sample of 60 participants with BED, differences of 10 ms posttraining could be detected with a power of ≥ 0.90. For baseline comparison, group differences of 20 ms could be detected for group sizes of BED: *n* = 60, NCG: *n* = 30 and OCG: *n* = 30 at a power of ≥. 90.

### Study procedure

For the group with BED, participation involved 13 appointments over approximately 5 months. The full schedule can be found in the additional files (Additional file 1); only the appointments relevant for addressing our research questions are described here. Prior to the first session, eligibility was assessed via a standardized phone screening. Eligible participants then attended a diagnostic interview, including the SCID-I, SCID-II, and EDE assessments, which determined final inclusion. After inclusion, the participants completed online questionnaires from home. A dot-probe paradigm (DPP) was administered the following week, starting with a standardized breakfast to ensure a consistent state of satiety, which could influence the processing of food stimuli [59, 60]. The control group participation ended after the baseline experiments. The participants with BED were randomly assigned to either AMT or the placebo training (AMT-P), which was completed in four sessions over 2–4 weeks. The DPP was repeated the following week, and eating pathology was reassessed via the EDE two weeks later. After the final assessment, the participants were informed of their group assignment, and those in the AMT-P group were offered AMT in the following weeks.

### Diagnostics

For eating disorder diagnosis, we used the German version of the EDE [56, 57], a widely used semistructured expert interview assessing the specific psychopathology of eating disorders. Only the diagnostic items were used to examine whether the DSM-5 criteria for BED, bulimia nervosa (BN) or anorexia nervosa were met. Weight and height were measured onsite. The German version of the SCID, a semistructured clinical interview to identify symptoms, syndromes and diagnoses of selected DSM-IV Axis 1 (SCID I) and Axis 2 (SCID II, personality disorders) diagnoses, was used to identify comorbid or previous mental disorders [58, 61, 62]. The interviews were tape recorded for quality control and reliability checks.

### Descriptive questionnaires

General eating pathology was assessed via self-reports using the Eating Disorder Examination-Questionnaire (EDE-Q; [63], German version [64]), which includes the four subscales Restraint (5 items), Eating Concern (5 items), Weight Concern (5 items) and Shape Concern (8 items). The four scales were combined into a total score that reflects the general eating disorder pathology. Cronbach’s α for the total score was excellent in our sample (Cronbach’s α = 0.96). Furthermore, the Dutch Eating Behaviour Questionnaire ( [65], German version [66]) was used to assess other aspects of eating behavior. The questionnaire contains 30 items loading on three subscales: external eating behavior (10 items, Cronbach’s α = 0.92), emotional eating behavior (10 items, Cronbach’s α = 0.97) and restrictive eating behavior (10 items, Cronbach’s α = 0.88).

In addition, depression severity (Beck Depression Inventory II [67, 68], Cronbach’s α = 0.95), the presence of restrained eating (Restraint Scale [69, 70], Cronbach’s α = 0.87), impulsivity (Barrat Impulsiveness Scale—short version [71, 72], Cronbach’s α = 0.82) and craving for food (Food Cravings Questionnaire—Trait, short version [73, 74], Cronbach’s α = 0.99) were assessed.

### DPP

**Fig. 1:**
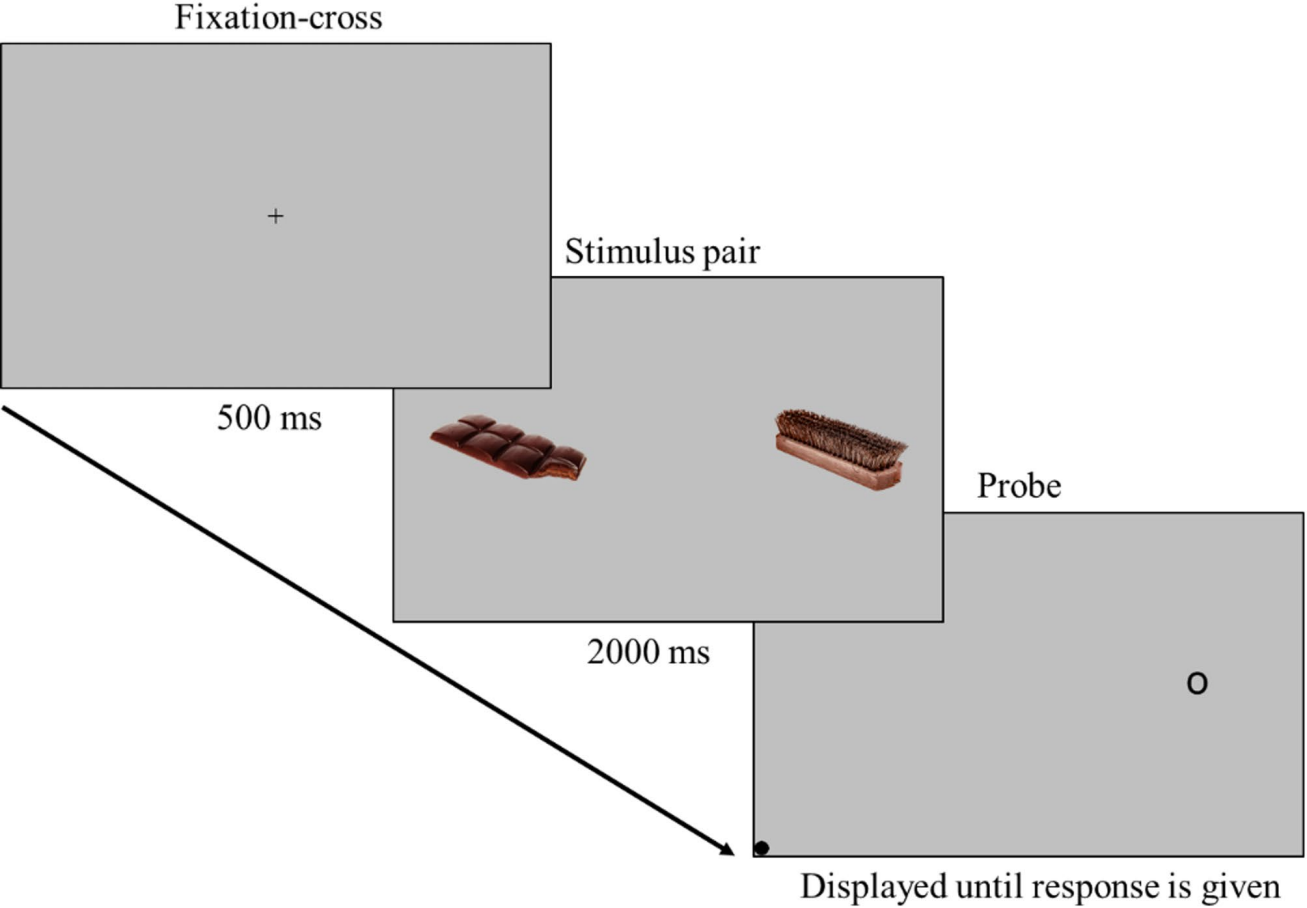
Visualization of trials in the dot–probe paradigm. Notes: The pictures presented here are for illustrative purposes only and were not used in the study

The DPP [75, 76] was used to assess selective attention processes. In the DPP, an affective stimulus and a neutral stimulus are presented simultaneously; participants respond to a probe that replaces one of them. Faster responses when the probe replaces the disorder-relevant stimulus (congruent trials) indicate an AB for these stimuli [11, 75]. The trials were presented on a 22” screen (1680 × 1050) at a distance of 70 cm. Presentation and recording were performed via Presentation software (Neurobeh. Systems, USA). Gaze data were collected at 250 Hz via an SMI eye tracker (Teltow, Germany) and recorded via IViewX. Each trial began with a 500 ms fixation cross, followed by a 2000 ms display of a stimulus pair (Fig. 1). A lateralized probe then appeared, and the participants indicated its position (left or right) by pressing a key. The stimuli included 20 critical image pairs (high-calorie food vs. musical instruments) and 10 filler nonfood image pairs. All image pairs were previously tested and matched for color, complexity, and brightness [12, 32]. Each pair appeared four times, resulting in 120 trials presented in pseudorandomized order with balanced probe locations. The Food-Craving-Questionnaire-State [73, 77] was administered at the beginning and end of the experiment to measure momentary craving. The questionnaire includes 15 items rated on a 5-point Likert scale. The internal consistency was excellent for all measurements (Cronbach’s α = .95−.96).

### AMT

For the AMT, 30 pairs of high-calorie food and neutral images were matched for color, complexity, brightness, and size. The images used were distinct from those used in the DPP. Each trial began with a 500 ms fixation on a central cross, followed by a 600 ms presentation of the image pair, displayed above and below the fixation cross. The participants indicated via keystroke which image was replaced by the probe. A total of 840 trials were conducted, with a 30-second break midway. During the first and last 120 trials, the probe replaced food or neutral images equally often. For the middle 600 trials, the probe replaced the neutral image in 90% of the cases, implicitly training participants to direct attention away from food stimuli [78]. Participants were told that the task was intended to influence reward sensitivity, while its actual aim of implicitly modifying attentional focus was not disclosed.

As a control condition, half of the participants with BED received the placebo version of the AMT. The AMT-P procedure mirrored the AMT, except that the probe replaced the neutral and food stimuli in a balanced manner.

### Data preprocessing

#### Preprocessing of eye-tracking data

Eye-tracking data were preprocessed via BeGaze 3.7 (Sensomotoric Instruments GmbH, 2017). Event detection was velocity-based, with saccades as primary events. The minimum saccade duration was set to 22 ms, and the peak velocity threshold was set to 40°/s. Fixations were defined using a minimum duration threshold of 100 ms [79]. Areas of interest (AOIs) were defined around each stimulus and categorized as either food or nonfood.

In further processing via Microsoft Access (2016), fixations were considered valid only if their dispersion was less than 100 px. Only the critical trials were used to calculate the AB. Datasets were excluded if more than 50% of critical trials lacked valid fixations (*n* = 1) or if participants lacked horizontal fixations beyond 200 ms from the center in more than 25% of critical trials (“starers” [79]). With fewer than 20 trials to calculate the AB indices, the measures would be highly biased by single trials and unrepresentative.

#### AB indices

##### Direction bias

Direction bias, the proportion of first fixations on food AOIs versus nonfood AOIs, was measured to detect facilitated attentional engagement toward food [11, 32]. Values above 0.5 indicate a direction bias toward food; values below 0.5 indicate a direction bias away from food.

##### Initial fixation duration

Early maintenance of attention was assessed through initial fixation duration [11, 32], which was calculated by summing fixation times on the first AOI until attention shifted elsewhere. The difference in the average initial fixation durations on food and nonfood AOIs across trials was used as the bias score, with positive values indicating a bias toward food [80].

##### Dwell time bias

Maintenance of attention and disengagement difficulties were assessed through gaze dwell time bias [11]. Dwell time bias, representing total fixation duration per category (food or nonfood AOIs), was calculated by summing valid fixations during the 2000 ms presentation, dividing by the number of trials with valid fixations, and calculating the difference between food and nonfood stimuli. Positive values indicate a bias toward food stimuli.

##### Dwell time bias variability

Dwell time bias variability was determined by computing the mean of the absolute differences between dwell time bias scores observed in consecutive trials [50].

#### Preprocessing of RT data

RTs were filtered via SPSS 28.0.0.0 by excluding incorrect responses, RTs < 200 ms or >2000 ms, or those exceeding 3 SDs from the individual mean [18], resulting in the exclusion of 2.16% of the trials.

#### RT bias

The mean RTs for congruent (probe replacing food) and incongruent trials (probe replacing nonfood) were used to calculate a difference score. Positive values indicate an AB toward food, and negative values indicate an AB away from food.

#### RT bias variability

RT bias variability was calculated by pairing each congruent trial with its nearest incongruent trial (and vice versa), within a maximal distance of five trials, and computing the difference in RTs (RT for the congruent trial minus RT for the incongruent trial). The mean of the absolute values of these difference scores was then taken as the variability index [35].

### Data analysis

Unless stated otherwise, all analyses were conducted via SPSS 28.0.0.0. The p value for significance was set to *p* < .05. Outliers were included in the data analysis. The assumptions were tested for the planned parametric analyses, and nonparametric alternatives were used when the assumptions were not met. Parametric analyses were repeated excluding outliers; the results are reported only if they differ in interpretation.

Sample characteristics were analyzed through descriptive statistics, and group differences were assessed through ANOVAs with subsequent post hoc tests, the nonparametric Kruskal-Wallis test with subsequent Dunn-Bonferroni or chi-square tests. Differences in the AB indices were calculated accordingly. Correlations between AB measures, craving, EDE-Q scores and binge eating frequency were calculated for the total sample and each group separately. P values were adjusted via the Holm-Bonferroni method [81].

Changes in AB indices following AMT or AMT-P were analyzed via mixed-model ANOVAs when assumptions were met, with group (AMT vs. AMT-P) as the between-subject factor and time (T0 [pre] vs. T1 [post]) as the within-subject factor. If the ANOVA assumptions were violated, we employed the F1-LD-F1 function from the nParLD package in R—a robust rank-based method for longitudinal data—using the nonparametric ANOVA-type statistic (ATS [82]). Change scores (T1-T0) were calculated for the AB indices, binge eating frequency, and cravings, and correlations were calculated.

## Results

### Sample description

**Fig. 2:**
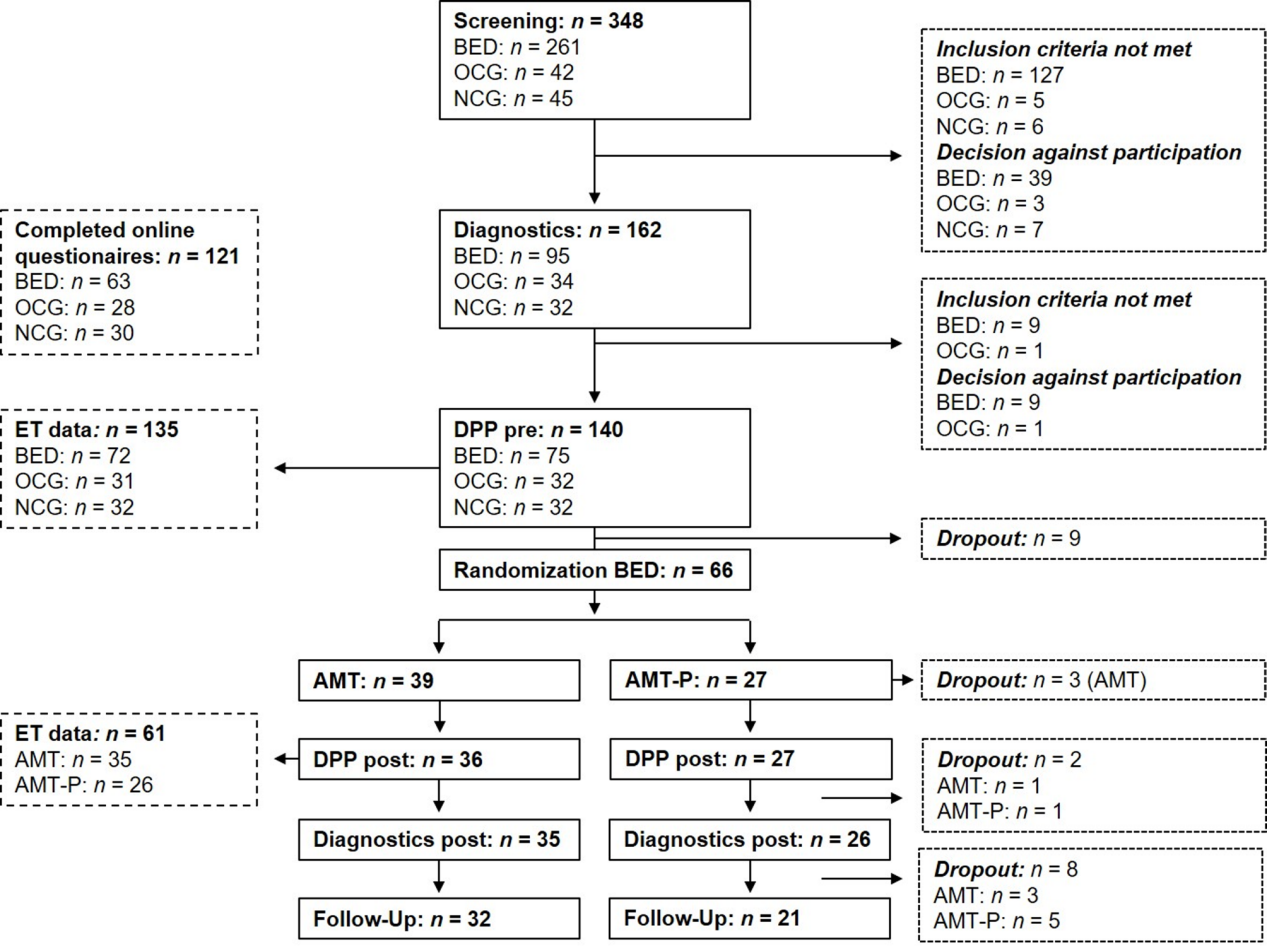
Flow of participants throughout the study. Some eye-tracking (ET) data were not available because of technical issues. BED = group with binge eating disorder; OCG = control group with overweight; NCG = control group with normal weight; DPP = dot probe paradigm; AMT = attention modification training; AMT-P = placebo version of the AMT

The participant flowchart is depicted in Fig. 2. Two subjects were excluded after re-evaluation of the diagnostic interviews^1^ (one lacked a BED diagnosis; one had bipolar disorder). For eye-tracking analyses, exclusions due to starers (*n* = 24) and poor data quality (*n* = 1) reduced the sample to 110 (BED: *n* = 65; NCG: *n* = 24; OCG: *n* = 21).

Sociodemographic and psychopathological data are summarized in Table 1. The group with BED differed from the control groups in all variables except sex; for most variables, the group with BED had higher scores than the control groups did (post hoc statistics are provided in Additional File 2). The intended age matching across groups was not fully achieved, as the NCG was younger than the group with BED; therefore, analyses including these group were controlled for age. In addition to BED, affective disorders were the most common SCID I diagnosis in the sample (NCG: *n* = 1, OCG: *n* = 3, BED: *n* = 20), followed by anxiety disorders (NCG: *n* = 1, OCG: *n* = 1, BED: *n* = 7).

**Table 1:** Baseline values for sociodemographics and overall psychopathology

	*n*	NCG *M (SD)*	OCG *M (SD)*	BED *M (SD)*	Test statistics	Sig. *p*	Post hoc
Age	135	28.03 (10.70)	34.03 (14.31)	38.96 (14.84)	χ^2^ (2) = 13.45	.001	NCG < BED
Sex	135				χ^2^ (2) = 3.31	.192	
Female		26	21	60			
Male		6	10	12			
(Comorbid) diagnosis [%]	135						
SCID I		6.3	16.1	38.9	χ² (2) = 14.30	< .001	NCG < BED
SCID II^a^		0	6.5	12.5	χ² (2) = 4.69	.077	
BMI	134^b^	21.81 (1.78)	28.50 (3.44)	29.65 (6.01)	χ^2^ (2) = 49.87	< .001	NCG < OCG = BED
EDE-Q	121^c^	1.34 (0.40)	1.67 (0.66)	3.80 (1.11)	χ^*2*^ (2) = 80.29	< .001	NCG = OCG < BED
DEB-Q							
Emotional	121^c^	1.65 (0.66)	1.51 (0.60)	3.77 (0.85)	χ^*2*^ (2) = 79.09	< .001	NCG = OCG < BED
External	121^c^	2.56 (0.62)	2.29 (0.72)	3.68 (0.63)	*F*(2, 118) = 57.74	< .001	NCG = OCG < BED
Restraint	121^c^	1.79 (0.60)	1.98 (0.59)	2.72 (0.72)	χ^*2*^ (2) = 37.04	< .001	NCG = OCG < BED
BDI II	121^c^	2.07 (3.41)	2.61 (4.36)	14.42 (10.54)	χ^*2*^ (2) = 56.00	< .001	NCG = OCG < BED
RS	121^c^	6.77 (3.28)	11.64 (4.16)	20.83 (4.51)	*F*(2, 118) = 129.69	< .001	NCG < OCG < BED
BIS-15	121^c^	31.10 (6.06)	27.04 (4.65)	33.02 (6.51)	χ^*2*^ (2) = 19.69	< .001	OCG < NCG = BED
FCQ-T-r	121^c^	20.87 (6.58)	20.79 (6.20)	63.84 (13.66)	χ^*2*^ (2) = 88.40	< .001	NCG = OCG < BED
FCQ-state	132^d^	1.29 (0.44)	1.35 (0.42)	2.03 (0.77)	χ^*2*^ (2) = 36.26	< .001	NCG = OCG < BED

The participants with binge eating disorder (BED, *n* = 72) were compared with those in the normal weight control group (NCG, *n* = 32) and the overweight control group (OCG, *n* = 31) via the Kruskal‒Wallis test, chi‒square test or ANOVAs. *p* < .05 indicate significant differences between groups. SCID = Structured Clinical Interview (SCID) for DSM-IV; BMI = Body-Mass Index; EDE-Q = Eating Disorder Examination Questionnaire; DEB-Q = Dutch Eating Behaviour Questionnaire; BDI II = Beck Depression Inventory II; RS = Restraint Scale; BIS-15 = Barrat Impulsiveness Scale-short version; FCQ-T-r = Food Cravings Questionnaire-Trait, short version; FCQ-state = Food Craving Questionnaire state

^a^Freeman‒Halton exact test was used, as expected cell frequencies were < 5

^b^NCG: *n* = 32, OCG: *n* = 31, BED: *n* = 71

^c^NCG: *n* = 30, OCG: *n* = 28, BED: *n* = 63

^d^NCG: *n* = 32, OCG: *n* = 31, BED: *n* = 69

### Baseline comparison of AB indices

**Fig. 3:**
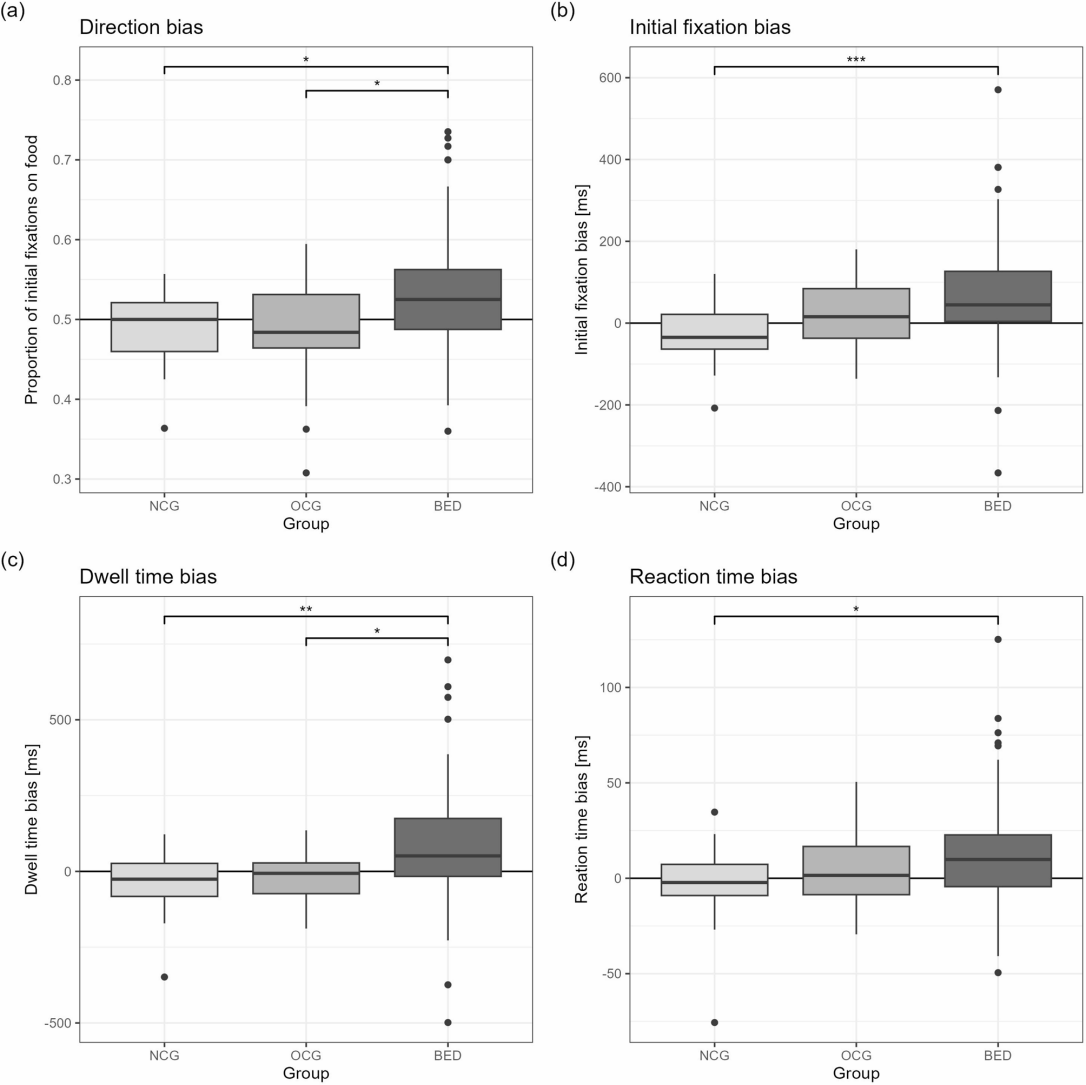
Boxplots to illustrate group differences in attentional bias indices. Please pay attention to the different scaling of the y-axis. A control group with normal weight (NCG, *n* = 24 for eye-tracking measures, *n* = 32 for reaction time bias), a control group with overweight (OCG, *n* = 21 for eye-tracking measures, *n* = 31 for reaction time bias) and a group with binge eating disorder (BED, *n* = 65 for eye-tracking measures, *n* = 71 for reaction time bias) are compared. Effect sizes for significant post hoc comparisons (Dunn-Bonferroni): **a** NCG – BED: *r* = .28, OCG – BED: *r* = .29; **b** NCG – BED: *r* = .38; **c** NCG – BED: *r* = .35, OCG – BED: *r* = .27; **d** NCG – BED: *r* = .27. * = *p* < .05, ** = *p* < .01, *** = *p* < .001 (Bonferroni corrected)

There were significant group differences (Kruskal-Wallis test) for direction bias (χ^2^(2) = 11.05, *p* = .004, *p*_*cor*_ = .008), initial fixation duration bias (χ^2^ (2) = 13.72, *p* = .001, *p*_*cor*_ = .004), dwell time bias (χ^2^ (2) = 13.67, *p* = .001, *p*_*cor*_ = .004) and RT bias (χ^2^(2) = 7.60, *p* = .022, *p*_*cor*_ = .022), which were followed by Dunn‒Bonferroni tests (Fig. 3; for further statistics, see Additional file 3). Compared with the NCG and OCG, the group with BED presented greater direction bias and dwell time bias, and greater initial fixation bias and RT bias than the OCG only.

Given the age differences between groups, rank-based ANCOVAs were conducted to assess whether age could account for group differences. The inclusion of age as a covariate did not alter the results for the eye-tracking measures. For RT bias, the group effect was no longer significant when age was included (*F*(2, 130) = 2.38, *p* = .097). The overall model remained significant (*F*(3, 130) = 3.56, *p* = .016, *R²* = 0.08, *R²*_*adj.*_ = 0.05), and age was not a significant predictor (*F*(1, 130) = 2.62, *p* = .108).

### Correlations between AB indices and eating pathology

Relationships between eating-disorder pathology and AB indices were assessed using Spearman’s rho. As shown in Table 2, all AB measures exhibited small‐to‐moderate positive correlations with eating‐disorder symptoms, except for RT bias with binge eating frequency and direction bias with craving, which were not statistically significant.

**Table 2:** Spearman’s rank correlation for relationships between attentional bias indices and eating pathology

	Reaction time bias				Direction bias				Initial fixation duration bias			Dwell time bias		
	*n*	ρ	*p*	*p*_cor_	*n* ^a^	ρ	*p*	*p*_cor_	ρ	*p*	*p*_cor_	ρ	*p*	*p*_cor_
EDE-Q	121	**0.23**	.012	.047	99	**0.30**	.002	.020	**0.38**	< .001	.001	**0.40**	< .001	.001
Number BEs	134	0.15	.076	.153	110	**0.33**	< .001	.005	**0.27**	.004	.022	**0.33**	< .001	.005
Craving	132	**0.23**	.009	.047	108	0.10	.306	.306	**0.28**	.003	.020	**0.25**	.010	.047

*p*_cor_ = corrected p values via Holm‒Bonferroni correction; EDE‒Q = Eating Disorder Examination Questionnaire; number BEs = sum of binge eating episodes over the last month

^a^ = *n* is the same for direction bias, initial fixation bias and dwell time bias

In the subgroup analyses, most of the correlations observed in the total sample decreased and lost significance. The relationship between craving and RT bias reached significance in both the OCG (ρ = 0.39, *n* = 31, *p* = .032) and the NCG (ρ = 0.39, *n* = 32, *p* = .013), but neither survived Holm-Bonferroni correction (*p*_*cor*_ = .859 and .371, respectively). In the group with BED, all correlations were small (ρs < .01-.25; see Additional File 4).

### Effects of AMT on AB

Only the group with BED underwent attentional training. We compared the AMT and the AMT-P on relevant baseline parameters (Table 3). The AMT-P group scored higher on the EDE-Q than did the AMT group. Otherwise, there were no significant differences between the groups.

**Table 3:** Comparison between the participants who completed attention modification training (AMT) and or placebo training (AMT-P)

	Time	*n*	AMT *M (SD)*	*n*	AMT-*P* *M (SD)*	Test Statistics	Sig. [*p*]
Age	T0	35	38.09 (15.26)	26	37.69 (14.87)	*U* = 429.00, *z* = − 0.38	.704
Gender	T0	35		26			.113
Male			3		6		
Female			32		20		
BMI	T0	35	28.38 (5.52)	26	30.72 (6.21)	*t*(59) = 1.56	.125
	T1	35	28.05 (5.90)	24	30.92 (5.64)		
	T2	29	28.34 (5.92)	19	32.16 (5.70)		
EDEQ total	T0	31	3.40 (1.09)	24	4.30 (0.99)	*t*(53) = 3.16	.003
BE 1 month	T0	35	13.14 (8.74)	26	11.08 (6.32)	*U* = 399.00, *z* = − 0.82	.413
	T1	35	6.71 (8.56)	24	8.21 (9.722)		
	T2	30	7.03 (9.41)	20	3.00 (2.90)		
Direction bias	T0	28	0.52 (0.07)	18	0.57 (0.08)	*t*(44) = 1.77	.084
	T1	28	0.52 (0.14)	18	0.54 (0.11)		
Initial fixation duration bias	T0	28	86.53 (148.49)	18	105.31 (137.73)	*U* = 245.00, *z* = − 0.16	.875
	T1	28	19.87 (140.45)	18	34.02 (141.15)		
Dwell time bias	T0	28	96.12 (223.57)	18	123.51 (209.41)	*U* = 245.00, *z* = − 0.16	.875
	T1	28	32.58 (201.47)	18	82.71 (171.09)		
Reaction time bias	T0	35	10.65 (30.37)	26	19.70 (34.20)	*U* = 424.00, *z* = 0.65	.651
	T1	35	7.29 (23.50)	26	6.79 (21.29)		
Craving	T0	35	2.13 (0.81)	26	1.93 (0.67)	*t*(59) = − 1.02	.313
	T1	35	1.66 (0.70)	26	1.76 (0.59)		

The participants were compared pre-training (T0) using Mann‒Whitney U tests or t tests. Additionally, scores for post-T1 training, as well as follow-up (T2), are reported, if present. p values < .05 indicate significant differences between groups. EDE-Q = Eating Disorder Examination Questionnaire; BE = Binge episodes

**Fig. 4:**
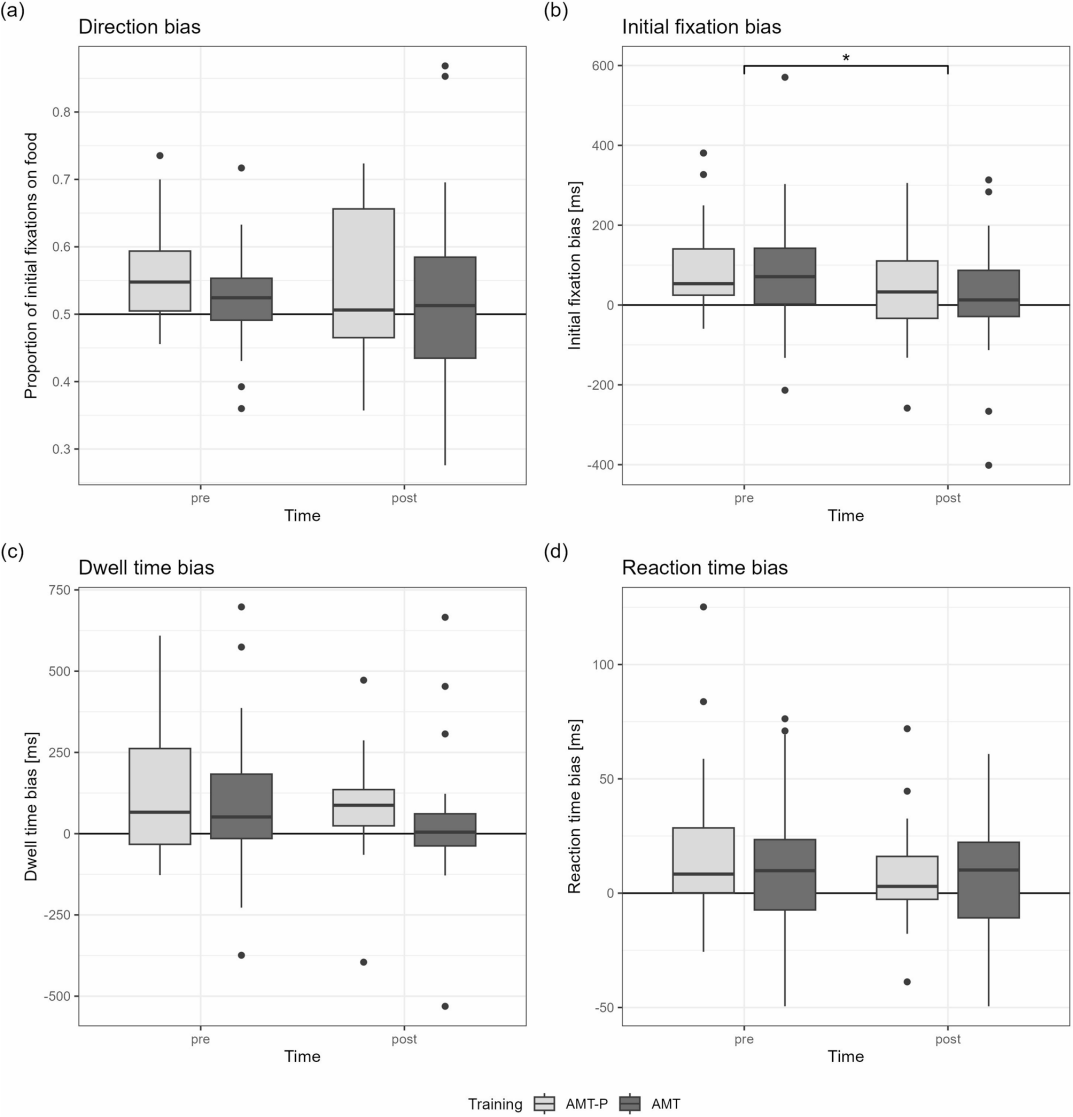
Differences in attentional bias pre and post attentional modification training (AMT) and placebo training (AMT-P). Please pay attention to the different scaling of the y-axis. Individuals with binge eating disorder were randomly assigned to AMT (*n* = 28 for eye-tracking measures, *n* = 35 for reaction time bias) or AMT-P (*n* = 18 for eye-tracking measures, *n* = 26 for reaction time bias). *p < .05

The expected interaction effects of time and training could not be found for any of the AB indices (*p* > .05; Fig. 4, for statistics see Additional file 5). Only for initial fixation duration bias was there a main effect of time, with scores decreasing from T0 to T1, Wilcoxon test: *z* = −2.24, *p* = .026, *n* = 46, *r* = 0.33 (Fig. 4).

### Correlations of the change scores

We examined correlations between changes in binge eating frequency and cravings and changes in AB indices^2^. Correlations (Spearman’s Rho) were small to moderate (Table 4). Only the correlation between changes in dwell time bias and craving was significant but did not survive Holm–Bonferroni correction (*p*_*cor*_ = .351).

**Table 4:** Nonparametric correlations (Spearman’s Rho) between changes before to after attentional modification training or placebo training

	Δ Binge episodes last month				Δ Craving			
	*n*	ρ	*p*	*p*_cor_	*n*	ρ	*p*	*p*_cor_
Δ Direction bias	45	− 0.02	.880	1	46	− 0.02	.880	1
Δ Initial fixation duration bias	45	0.16	.291	1	46	0.22	.141	0.987
Δ Dwell time bias	45	0.09	.578	1	46	0.30	.044	0.351
Δ Reaction time bias	59	− 0.01	.955	1	61	− 0.10	.460	1

p values were corrected using the Holm-Bonferroni method

### Dynamic AB indices

Dwell time bias variability at baseline did not differ between groups, was not correlated with eating pathology except for a negative correlation with binge eating frequency in the group with BED (ρ = − 0.27, *n* = 65, *p* = .032), and did not change during AMT or AMT-P (Additional file 7).

RT bias variability was significantly correlated with initial fixation duration bias (ρ = 0.22, *n* = 109, *p* = .022), and correlations for other AB measures were small and not significant (ρ < 0.16, *p* > .05). The groups differed in RT bias variability (Kruskal-Wallis test; χ^2^(2) = 14.24, *p* < .001), with higher scores for the group with BED (*Mdn* = 79.17) than for the NCG (*Mdn* = 60.87; *z* = 3.56, *p* < .001, *p*_*cor*_ = 0.001) but not significantly higher than those for the OCG (*Mdn* = 68.77, *z* = 2.28, *p* = .022, *p*_*cor*_ = 0.067). The group difference remained significant when accounting for age (group: *F*(2,130) = 0.397, *p* = .021), with age itself also being a significant factor (*F*(1,130) = 13.40, *p* < .001). RT bias variability was correlated with EDE-Q (ρ = 0.26, *n* = 121, *p* = .032), binge eating frequency (ρ = 0.19, *n* = 134, *p* = .027), and craving (ρ = 0.25, *n* = 132, *p* = .004) in the whole sample. The correlations were smaller in the subgroups except for a significant negative correlation with binge eating frequency in the group with BED (ρ = − 0.33, *n* = 71, *p* = .005; follow-up analyses in Additional file 8). There was no significant interaction effect of training group and time on RT bias variability (*ATS* = 0.24, *p* = .622), but there was a main effect of time (*ATS* = 7.28, *p* = .007), with scores decreasing from T0 (*Mdn* = 78.18) to T1 (*Mdn* = 69.75; Wilcoxon test: *z* = 9.59, *p* = < 0.001, *n* = 61, *r* = .31). Changes in RT bias variability were not significantly correlated with changes in craving (ρ = 0.10, *n* = 61, *p* = 460) or binge eating frequency (ρ = − 0.10, *n* = 59, *p* = .434).

### Further exploratory analyses

Since most AB indices did not significantly change over time, we analyzed changes in the dwell time on food and nonfood images, as well as the RTs, prior to computing the bias indices. There were no interactions of group × time (*p* > .05; Additional file 9), but dwell time decreased from T0 to T1 (Wilcoxon test: *z* = − 2.60, *p* = .009, *r* = .38), as did RTs (*z* = − 3.13, *p* = .002, *r* = .40; Table 5). Finally, we analyzed whether training had a significant effect on AB toward food with only participants with an AB toward food at baseline included. No significant interaction effects were observed, but the main effect of time was significant for all bias indices (Additional file 9). The AB scores decreased from T0 to T1 for direction bias (Wilcoxon test: *z* = − 2.45, *p* = .014, *r* = .45), initial fixation duration bias (*z* = − 2.77, *p* = .006, *r* = .46), dwell time bias (*z* = − 2.95, *p* = .003, *r* = .52) and RT bias (*z* = − 3.32, *p* = < 0.001, *r* = .59; Table 5).

**Table 5:** Descriptives for exploratory group comparisons pre (T0) to post (T1) attention modification training (or placebo)

Sample	Variable	*n*	T0		T1	
			*M (SD)*	*Mdn*	*M (SD)*	*Mdn*
Whole sample	Gaze dwell time	46				
	Food		540.34 (216.72)	540.77	433.29 (215.15)	425.26
	Nonfood		433.50 (168.09)	452.50	381.09 (191.05)	381.56
	Reaction time	61				
	Congruent		494.87 (104.77)	484.31	459.12 (75.12)	448.13
	Incongruent		509.37 (114.76)	486.92	466.20 (78.04)	450.90
Existing attentional bias toward food at baseline	Direction bias	30	0.58 (0.06)	0.55	0.53 (0.11)	0.51
	Initial fixation bias	36	136.72 (128.75)	89.59	44.24 (135.89)	25.33
	Dwell time bias	32	195.21 (193.03)	108.87	85.40 (180.05)	38.63
	Reaction time bias	42	28.62 (27.72)	17.97	7.48 (22.82)	4.94

## Discussion

In the present study, we examined attentional processes toward food in individuals with BED and evaluated whether a computer-assisted AMT could robustly modify these processes. Consistent with prior research, individuals with BED exhibited a baseline AB toward food-related stimuli compared with healthy controls; however, our AMT protocol did not demonstrate evidence of producing sustained changes in attentional processing.

Our baseline assessments largely confirmed our hypotheses: individuals with BED exhibited an AB toward food cues, which is consistent with prior findings of heightened food-related attention in BED [11]. Consistent with prior studies, this bias was most pronounced in dwell time, which reflects difficulties disengaging from food stimuli [14–16, 79] and is also evident in direction bias, indicative of initial attentional allocation to food stimuli [12, 13]. No difference was observed between the NCG and the OCG in either of the two measures, supporting meta-analytic findings that these measures are linked to eating pathology rather than BMI [34].

In contrast, initial fixation duration bias was significantly greater in the group with BED than in the NCG, but—contrary to our hypotheses—did not differ significantly from the OCG. This suggests that weight status may play a secondary role in the development of AB: Because obesity is usually linked to increased food intake, sensitization of neural pathways and a corresponding amplification of AB toward food could arise even without binge eating [26, 83]. However, since the NCG and OCG did not differ in any AB indices, elevated BMI or overeating alone appears insufficient to produce a group-level bias. Baseline RT bias is not interpreted here, given its documented low reliability [84, 85] and the fact that any group differences became non‐significant after controlling for age.

AB levels demonstrated small-to‐moderate positive correlations with eating disorder severity in the full sample. Contrary to our hypothesis, this relationship was not stronger in the group with BED; the correlations diminished when analyzed within subgroups. This pattern suggests that these correlations are driven primarily by group differences rather than direct associations of AB toward food with EDE-Q scores, the frequency of binge eating episodes, or craving. Thus, our findings align with previous evidence indicating only modest relationships between AB toward food and measures of eating disorder pathology such as craving [86].

In addition to the findings regarding mean AB measures, this is the first study to compare dynamic AB indices among individuals with BED and healthy controls. Prior research has shown that the AB fluctuates within participants from trial to trial [35] and indicates that greater variability in the AB is linked to impaired attentional control [87, 88], emotional dysregulation [89, 90], and motivational conflict toward affective stimuli [91]. Because individuals with BED exhibit deficits in attentional control [92], heightened emotional dysregulation [93], and ambivalent motivation toward high-calorie foods [94], we expected that AB variability would be elevated in the group with BED relative to both control groups.

Surprisingly, dwell time bias variability did not differ among the groups and was unrelated to both EDE-Q scores and craving, showing only a negative association with binge-eating frequency (to be discussed below). Moreover, dwell time bias variability was uncorrelated with most mean AB indices, except for a modest relationship with initial fixation duration bias. Therefore, in the present sample and using our adopted calculation, dwell time bias variability appears mostly unrelated to BED symptomology. Notably, no standardized method exists for deriving dynamic, gaze-based AB metrics in the DPP. Here, we applied the same approach used in our earlier work [50], which considers both the amplitude and frequency of attentional shifts toward and away from affective stimuli. Alternative algorithms have been proposed (e.g [33, 95, 96])^3^, and future research is needed to evaluate and compare the reliability of these different calculation methods.

RT bias variability has demonstrated adequate reliability in previous studies [35, 85], although more recent work suggests this may be driven primarily by its strong association with overall RT variability [97]. In the current sample, participants with BED exhibited greater RT bias variability than did those in the NCG, yet no significant difference emerged between the group with BED and the OCG. Findings indicating a link between BMI and RT bias variability may account for this nonsignificant difference [33, 95]. Furthermore, our analyses revealed that age was associated with RT bias variability. Considering that both higher BMI and older age are related to diminished executive functioning [98, 99], these results lend support to the idea that RT bias variability may be tied to executive functioning. However, the precise mechanisms underpinning food-specific RT bias variability remain unclear [100].

Notably, RT bias variability distinguished the group with BED from the NCG even after controlling for BMI and age, and it correlated positively with EDE-Q scores, craving, and binge eating frequency, suggesting a unique link to eating pathology. Interestingly, we also found a negative correlation between RT bias variability and binge eating frequency in the group with BED, mirroring the inverse relationship observed with dwell time bias variability. Follow-up analyses revealed that participants with more frequent binge eating episodes did not display fewer attentional shifts per se but rather had smaller mean reaction time biases in both directions in each trial. A similar (though nonsignificant) trend was observed for trial-level dwell time bias. These findings indicate that individuals with higher frequency of binge eating episodes exhibit smaller differences in responding to food versus non-food stimuli. Given our relatively long stimulus duration (2000 ms), it is also possible that those with more binge episodes engage in rapid, within-trial shifts between engagement and avoidance—patterns not captured by our trial-level metrics. Future studies should replicate these exploratory findings and test these hypotheses by using shorter stimulus presentations or by tracking intra‐trial dynamic gaze patterns.

After establishing the prerequisite for successful AMT—that a baseline AB toward food was present in our sample—we examined whether AB improvements observed during AMT [50] would generalize to an alternative stimulus set and modified task parameters. Contrary to our hypotheses, no differential effects emerged: although initial fixation duration bias and RT bias variability decreased from pre- to post‐intervention, these reductions were equivalent in both the AMT and AMT‐P groups.

One possible explanation is that implicit attentional training may have occurred in both groups. Individuals with BED exhibited a baseline AB, focusing more frequently and longer on food stimuli. Training with balanced contingencies, such as in the AMT-P, may already promote more balanced gaze behavior [101]. This is supported by findings that changes in all four mean AB indices were observed when analyses included only participants with a preexisting AB for food. In line with precision therapy and the multifactorial etiology of BED [102, 103], future studies should consider applying attentional training specifically for those individuals with BED, whose AB toward food is of large magnitude to test its effects on eating pathology in this specific subgroup.

Notably, other contingency-independent mechanisms may have influenced the outcomes in both groups. The total dwell times and RTs decreased from T0 to T1. This suggests a practice effect, where participants became more efficient at the task, spending less time dwelling at the images and responding more quickly. Furthermore, the training could have enhanced top-down attentional control through repeated practice of attentional training tasks amid exposure to task-irrelevant cues [54, 104]. The observed reduction in RT bias variability and initial fixation duration bias could both reflect improvements in attentional control, as previous research has linked lower RT variability [100] and enhanced disengagement from stimuli [105, 106] to stronger top-down regulatory processes.

Overall, the absence of sustained group differences indicates that the reductions in direction bias, dwell time bias, and dwell time bias variability observed during AMT [50] did not persist over time or generalize to alternative contexts. These findings suggest that training effects are highly context-specific—restricted to the exact parameters of the AMT sessions, which differed from the pre-post assessments in stimulus orientation (vertical vs. horizontal), stimulus set, and presentation duration. If such narrow specificity is accurate, it casts doubt on the ecological validity of the AMT protocol, indicating limited potential to influence attentional processes in real-world settings.

Alternatively, attentional processes may not have been implicitly trained during AMT. Instead, participants may have gained explicit knowledge of the contingencies and consequently enhanced their task performance by consciously directing attention toward neutral stimuli [107, 108]. Although this explicit strategy would manifest as a reduction in AB toward food within the training context, it would fail to generalize to a DPP with balanced contingencies, as the strategy would no longer be advantageous and therefore abandoned. Grafton et al. (2014) suggested that while explicit awareness of contingencies can indeed modify attentional selectivity, such explicit strategies impede the generalization to clinical symptoms [107].

The lack of generalization of attentional-process changes likely accounts for the absence of differential improvements in eating pathology, despite initial AMT-induced alterations in AB [50]. Both groups exhibited improvements in eating pathology, which may reflect concurrent changes in AB components across conditions. However, correlations between AB changes and reductions in binge eating frequency and craving were minimal; only the association between dwell time bias change and craving reached a moderate magnitude. Thus, alternative explanations warrant consideration. Repeated exposure to food cues in the absence of binge eating opportunities may have produced habituation or expectancy‐violation effects [109]. Indeed, exposure and response prevention interventions have demonstrated efficacy in reducing binge eating behaviors [110]. Moreover, study participation and repeated diagnostic assessments may have prompted self‐directed behavioral changes. Finally, expectancy effects or regression to the mean may have contributed to the observed reductions in eating pathology.

## Limitations

Several limitations warrant consideration. First, the AMT and AMT-P groups differed in terms of baseline eating‐disorder severity, with the AMT‐P cohort exhibiting higher EDE‐Q scores. In addition, unequal group sizes, stemming from sample‐size adjustments made in response to COVID‐19-related recruitment challenges, may have affected the statistical power and biased group comparisons.

Furthermore, the exclusion of starers reduced the effective sample size. Specifically, in the baseline measurements, approximately 15% of the datasets were excluded from the eye-tracking analyses, increasing to 18% for the pre-post comparisons. Although excluding these participants improved the accuracy of the AB measurements, it diminished the statistical power. Future studies should emphasize ensuring that participants direct their gaze toward stimuli rather than relying on peripheral vision. Incorporating tasks that require recognition of previously viewed images could enhance participant engagement without explicitly instructing fixation.

Moreover, the considerable variance observed in our variables further affected the statistical power. The simulation-based power analysis conducted prior to data collection assumed lower variance than was actually observed for the AB indices, potentially leading to an underestimation of the required sample size.

## Conclusion

Our findings confirm previous reports that individuals with BED exhibit altered attentional processes toward food stimuli compared with healthy controls. Moreover, this study is the first to demonstrate differences in RT bias variability between a group with BED and a NCG. Our results further indicate that although attentional processes can change during an AMT based on the DPP, these changes might not generalize beyond the specific training context. Instead, we observed nonspecific improvements in AB measures toward food unrelated to group allocation. Thus, while modifying AB appears feasible, further research into the underlying active mechanisms of AMT is essential to develop targeted and effective interventions for patients with BED.

## Supplementary Information

Below is the link to the electronic supplementary material.

Supplementary Material 1. File 1: Overall schedule for participation. File 2: Post hoc tests for comparisons of socio-demographic and psychopathological data. File 3: Descriptives and statistics for AB indices at baseline. File 4: Correlations of AB indices with eating pathology in subgroups. File 5: Effect of AMT on craving. File 6: Effect of AMT on craving. File 7: nalyses vor dwell time bias variability. File 8: Follow-up analyses for variability indexes in the group with BED. File 9: Additional statistics for exploratory analyses.

## Data Availability

The datasets used and analysed during the current study are available from the corresponding author on reasonable request.

## Footnotes

1 All other diagnostic decisions were confirmed.

2 Both AMT and AMT-P showed a decrease in number of binge eating episodes [50] and craving (Additional file 6).

3 Reanalysis using an alternative metric (standard deviation) did not change the results.

## Abbreviations

AB: Attentional bias
AOI: Area of interest
AMT: Attentional modification training
AMT-P: Attentional modification training – placebo
BED: Binge eating disorder
BN: Bulimia nervosa
DPP: Dot probe paradigm
DSM: Diagnostic and Statistical Manual of Mental Disorders
EDE: Eating disorder examination
EDE-Q: Eating disorder examination – questionnaire
IST: Incentive-sensitization theory
NCG: Control group with normal weight
OCG: Control group with overweight
RT: Reaction time
SCID: Structured Clinical Interview for DSM Disorders
