# Supplementary material for: Attentional bias toward food in binge eating disorder: baseline differences and the limits of attention modification training
Source: J Eat Disord. 2025 Nov 5;13:249. doi: 10.1186/s40337-025-01450-4 (PMC12590676; doi:10.1186/s40337-025-01450-4)
Supplement: Supplementary file 1 — Supplementary Material 1. File 1: Overall schedule for participation. File 2: Post hoc tests for comparisons of socio-demographic and psychopathological data. File 3: Descriptives and statistics for AB indices at baseline. File 4: Correlations of AB indices with eating pathology in subgroups. File 5: Effect of AMT on craving. File 6: Effect of AMT on craving. File 7: nalyses vor dwell time bias variability. File 8: Follow-up analyses for variability indexes in the group with BED. File 9: Additional statistics for exploratory analyses. [file 40337_2025_1450_MOESM1_ESM.docx]

**Additional file 1**

*Overall schedule for participation*


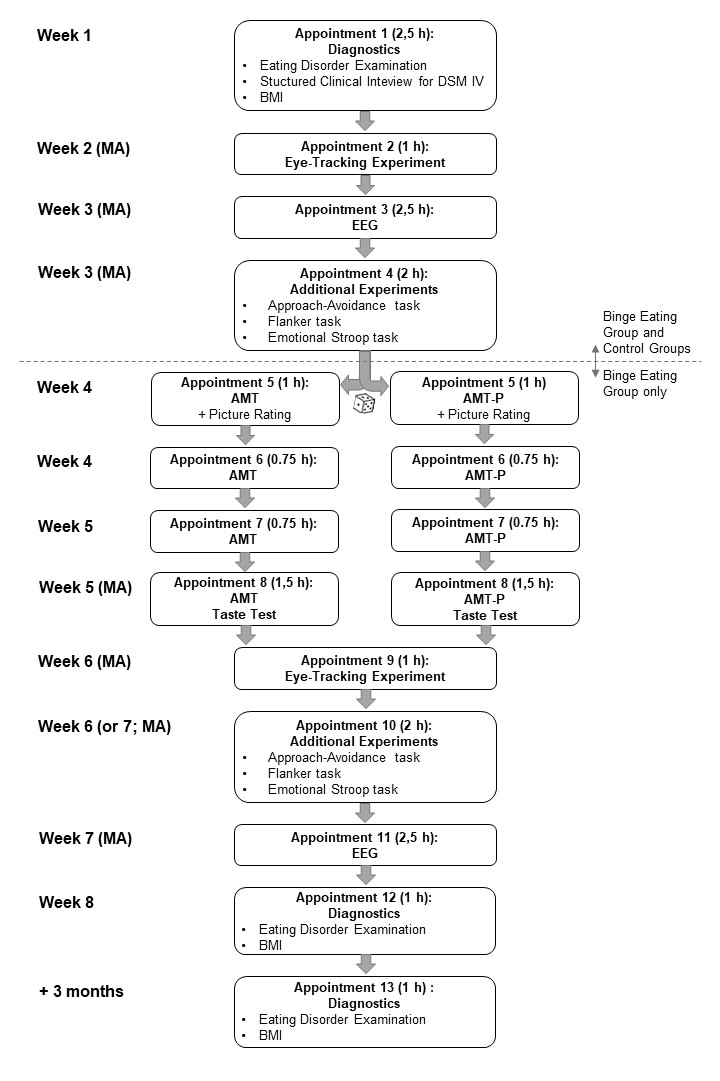


*Note.* AMT = Attention modification training; AMT-P = AMT placebo; MA = morning appointment. MAs were scheduled in the morning (usually starting between 8:00 and 8:30 a.m.) and included a standardized breakfast. The intervals between appointments were adhered to as far as possible; in some cases there were deviations from the schedule due to illness or scheduling conflicts.

**Additional file 2**

Post hoc tests for comparisons of socio-demographic and psychopathological data in the group with binge eating disorder, and control groups with normal weight and overweight for non-parametric analyses (Table AF 2.1) and parametric analyses (Table AF 2.2).

**Table AF 2.1**

*Pairwise comparisons (Dunn-Bonferroni tests) for the descriptive variables with significant differences found with Kruskal-Wallis tests*

|  | Comparison | Test statistics | SE | Standard test statistics | Sig. [*p*] | Corrected Sig. [*p*] |
| --- | --- | --- | --- | --- | --- | --- |
| Age^a^ | OCG - NCG | 18.55 | 9.85 | 1.88 | .060 | .179 |
|  | BED – NCG | **30.33** | 8.30 | 3.65 | <.001 | .001 |
|  | BED - OCG | 11.78 | 8.39 | 1.40 | .160 | .481 |
| BMI^a^ | OCG - NCG | **53.72** | 9.79 | 5.49 | <.001 | <.001 |
|  | BED – NCG | **56.29** | 8.27 | 6.81 | <.001 | <.001 |
|  | BED - OCG | 2.57 | 8.36 | 0.31 | .759 | 1 |
| EDE-Q^a^ | OCG - NCG | 12.19 | 9.22 | 1.32 | .186 | .558 |
|  | BED – NCG | **62.44** | 7.78 | 8.03 | <.001 | <.001 |
|  | BED - OCG | **50.25** | 7.97 | 6.31 | <.001 | <.001 |
| DEB-Q Emotional | OCG - NCG | 4.63 | 9.21 | 0.50 | .616 | 1 |
|  | BED – NCG | **54.39** | 7.77 | 7.00 | <.001 | <.001 |
|  | BED - OCG | **59.02** | 7.96 | 7.41 | <.001 | <.001 |
| DEB-Q Restraint | OCG - NCG | 8.15 | 9.20 | 0.89 | .376 | 1 |
|  | BED – NCG | **42.31** | 7.77 | 5.45 | <.001 | <.001 |
|  | BED - OCG | **34.17** | 7.96 | 4.30 | <.001 | <.001 |
| BDI II^a^ | OCG - NCG | 2.72 | 9.13 | 0.30 | .766 | 1 |
|  | BED – NCG | **48.60** | 7.71 | 6.30 | <.001 | <.001 |
|  | BED - OCG | **45.88** | 7.89 | 5.81 | <.001 | <.001 |
| BIS-15 | OCG - NCG | **26.11** | 9.20 | 2.84 | .005 | .014 |
|  | BED – NCG | 9.14 | 7.77 | 1.18 | .239 | .718 |
|  | BED - OCG | **35.24** | 7.95 | 4.43 | <.001 | <.001 |
| FCQ-T-r^a^ | OCG - NCG | 0.78 | 9.21 | 0.08 | .933 | 1 |
|  | BED – NCG | **59.60** | 7.78 | 7.66 | <.001 | <.001 |
|  | BED - OCG | **60.37** | 7.96 | 7.58 | <.001 | <.001 |
| FCQ-state | OCG - NCG | 5.37 | 9.59 | 0.56 | .575 | 1 |
|  | BED – NCG | **42.39** | 8.14 | 5.21 | <.001 | <.001 |
|  | BED - OCG | **37.02** | 8.23 | 4.50 | <.001 | <.001 |

*Note*. Participants with binge eating disorder (BED) are compared to a control group with normal weight (NCG) and a control group with overweight (OCG) using the Dunn-Bonferroni test for pairwise comparisons. P-Values < .05 indicate differences between groups. EDE-Q = Eating Disorder Examination Questionnaire; DEB-Q = Dutch Eating Behaviour Questionnaire; BDI = Beck Depression Inventory II; RS = Restraint Scale; FCQ-T-r = Food Cravings Questionnaire—Trait, short version; SE = standard error; Sig. = significance,

^a^ Games-Howell Test was used, as variance homogeneity was not given

**Table AF 2.2**

*Pairwise comparisons for the descriptive variables with significant differences found with ANOVA*

|  | Comparison | Difference | SE | Sig. [*p*]. | 95%-CI | |
| --- | --- | --- | --- | --- | --- | --- |
|  |  |  |  |  | Lower limit | Upper limit |
| DEB-Q External | OCG - NCG | -0.28 | 0.17 | .237 | -0.68 | 0.13 |
|  | BED – NCG | 1.12 | 0.14 | <.001 | 0.78 | 1.46 |
|  | BED - OCG | 1.40 | 0.15 | <.001 | 1.05 | 1.75 |
| RS total | OCG - NCG | 4.88 | 1.09 | <.001 | 2.28 | 7.47 |
|  | BED – NCG | 14.06 | 0.92 | <.001 | 11.87 | 16.25 |
|  | BED - OCG | 9.18 | 0.94 | <.001 | 6.94 | 11.42 |

*Note.* Participants with Binge Eating Disorder (BED) are compared to a control group with normal weight (NCG) and a control group with overweight (OCG) using the Tukey-Kramer post-hoc test for pairwise comparison. *p* < .05 indicate differences between groups. DEB-Q = Dutch Eating Behaviour Questionnaire; RS = Restraint Scale; SE = standard error; Sig. = significance; CI = confidence interval

**Additional file 3**

Table AF 3.1 contains the mean and median for the different attentional bias indices at baseline, for better retracing of the analyses. Table AF 3.2 shows the post hoc tests for the group differences.

**Table AF 3.1**

*Means (M), standard deviations (SD) and Median of attentional bias indices at baseline*

|  | Control group with normal weight | | Control group with overweight | | Group with binge eating disorder | |
| --- | --- | --- | --- | --- | --- | --- |
|  | *M (SD)* | *Mdn* | *M (SD)* | *Mdn* | *M (SD)* | *Mdn* |
| Direction bias | .49 (.05) | .50 | .48 (.07) | .48 | .54 (.08) | .53 |
| Initial fixation duration bias | -19.39 (83.57) | -34.84 | 22.50 (84.06) | 15.63 | 74.45 (140.17) | 44.67 |
| Dwell time bias | -34.74 (98.95) | -25.69 | -13.17 (84.42) | -6.42 | 84.86 (202.58) | 51.11 |
| Reaction time bias | -2.48 (19.17) | -2.23 | 6.07 (20.46) | 1.51 | 13.31 (30.45) | 9.85 |

**Table AF 3.2**

*Pairwise comparisons (Dunn-Bonferroni tests) for the attentional bias indices*

|  | Comparison | Test statistics | SE | Standard test statistics | Sig [*p*]. | Corrected Sig. [*p*]. | Effect size [*r*] |
| --- | --- | --- | --- | --- | --- | --- | --- |
| Direction bias | OCG – NCG | -0.53 | 9.53 | -0.06 | .955 | 1 | .01 |
|  | BED – NCG | 20.84 | 8.01 | 2.60 | .009 | .028 | .28 |
|  | BED – OCG | 20.30 | 7.62 | 2.67 | .008 | .023 | .29 |
| Initial fixation duration bias | OCG – NCG | 14.26 | 9.53 | 1.50 | .135 | .404 | .22 |
|  | BED – NCG | 27.62 | 7.62 | 3.62 | <.001 | .001 | .38 |
|  | BED – OCG | 13.36 | 8.01 | 1.67 | .095 | .285 | .18 |
| Dwell time bias | OCG – NCG | 4.79 | 9.53 | 0.50 | .615 | 1 | .07 |
|  | BED – NCG | 24.89 | 7.62 | 3.27 | .001 | .003 | .35 |
|  | BED – OCG | 20.10 | 8.01 | 2.51 | .012 | .036 | .27 |
| Reaction time bias | OCG – NCG | 11.87 | 9.79 | 1.21 | .225 | .676 | .15 |
|  | BED – NCG | 22.48 | 8.27 | 2.72 | .007 | .020 | .27 |
|  | BED – OCG | 10.61 | 8.36 | 1.27 | .204 | .612 | .13 |

*Note*. Participants with binge eating disorder (BED) are compared to a control group with normal weight (NCG) and a control group with overweight (OCG). SE = standard error; Sig. = significance

**Additional file 4**

*Spearman’s rank correlation for relationships between attentional bias indices and eating pathology for control groups with normal weight (NCG ), overweight (OCG) and a group with binge eating disorder (BED)*

|  |  | Reaction time bias | | | Direction bias | | | Initial fixation duration bias | | | Dwell time bias | | |
| --- | --- | --- | --- | --- | --- | --- | --- | --- | --- | --- | --- | --- | --- |
|  |  | NCG | OCG | BED | NCG | OCG | BED | NCG | OCG | BED | NCG | OCG | BED |

| EDEQ | ρ | .11 | .23 | -.17 | .05 | .00 | .03 | .13 | .36 | .16 | .15 | .08 | .21 |
| --- | --- | --- | --- | --- | --- | --- | --- | --- | --- | --- | --- | --- | --- |
|  | *n* | 30 | 28 | 69 | 22 | 19 | 58 | 22 | 19 | 58 | 22 | 19 | 58 |
| Number BEs | ρ | .^a^ | .^a^ | -.14 | .^a^ | .^a^ | .12 | .^a^ | .^a^ | -.02 | .^a^ | .^a^ | .05 |
|  | *n* | 32 | 31 | 71 | 24 | 21 | 65 | 24 | 21 | 65 | 24 | 21 | 65 |
| Craving | ρ | .43* | .39* | <.01 | .12 | -.34 | .02 | .08 | .11 | .24 | .01 | .05 | .20 |
|  | *n* | 32 | 31 | 71 | 24 | 21 | 65 | 24 | 21 | 65 | 24 | 21 | 65 |

*Note.* EDE-Q = Eating Disorder Examination Questionnaire; number BEs = sum of binge eating episodes in the last month

^a^ Correlations could not be calculated as no BEs occurred in the control groups

*p < .05 (uncorrected)

**Additional file 5**

*Statistics for the effect of training over time*

| Mixed-Model ANOVA | *n* | Effect | *F* | df | Sig. [*p*] | η^2^ |
| --- | --- | --- | --- | --- | --- | --- |
| Direction bias | 46 | Time | 0.37 | 1, 44 | .548 | .01 |
|  |  | Group | 1.60 | 1, 44 | .212 | .04 |
|  |  | Time*Group | 0.26 | 1, 44 | .611 | .01 |
|  |  |  |  |  |  |  |
| npar | *n* | Effect | ATS | df | Sig. [*p*] |  |
| Initial fixation duration bias | 46 | Time | 5.52 | 1 | .019 |  |
|  |  | Group | 0.15 | 1 | .702 |  |
|  |  | Time*Group | 0.01 | 1 | .924 |  |
| Dwell time bias | 46 | Time | 0.65 | 1 | *.*421 |  |
|  |  | Group | 1.75 | 1 | .185 |  |
|  |  | Time*Group | 2.47 | 1 | .116 |  |
| Reaction time bias | 61 | Time | 1.38 | 1 | .240 |  |
|  |  | Group | 0.01 | 1 | .928 |  |
|  |  | Time*Group | 0.44 | 1 | .507 |  |

*Note*. 2 (training condition) × 2 (time) analysis via mixed-model ANOVAs or a nonparametric rank-based procedure (npar)

**Additional file 6**

The effect of AMT on craving was examined using R's “nparLD” package, applying the robust rank-based F1-LD-F1 function for longitudinal data. ANOVA-type statistic (ATS) was used. Group (AMT vs. AMT-P) served as the between-subject factor, and time (T0 [pre] vs. T1 [post]) as the within-subject factor. There was no significant time-by-group interaction (*ATS* = 2.69, *df =* 1, *n =* 61, *p* = .101). However, the main effect of time was significant (*ATS* = 11.13, *df =* 1, *n =* 61, *p* < .001), with lower craving scores at T1 (*Mdn* = 1.67) compared to T0 (*Mdn* = 2.06; Wilcoxon test: *z* = –3.14, *p* = .002, *r* = .40)

**Additional file 7**

Dwell time bias variability was significantly correlated with initial fixation duration bias (ρ = .25, *n* = 110, *p* = .008), correlations for other AB measures were small and not significant (ρ < |.14|, p >.05). The group with BED (*Mdn* = 539.04), NCG (*Mdn* = 503.92) and OCG (*Mdn* = 489.25) did not differ in terms of dwell time bias variability (Kruskal‒Wallis test; χ^2^(2) = 1.21, *p* = .547), irrespective of age. Dwell time bias was not significantly correlated with the EDE-Q score or the number of BEs or cravings in the whole sample (ρ < |.14|, p >.05), but there was a negative correlation with the number of BEs in the group with BED (ρ = -.27, *n* = 65, *p* = .032). There was no significant interaction effect of group or time on dwell time bias variability (mixed ANOVA; *F*(1,44) <0.01, *p* = .997, η^2^ <.01), nor was there a main effect of time (*F*(1,44) = 0.49, *p* = .488, η^2^ = .01). Changes in dwell time bias variability were not correlated with changes in craving (ρ = .14, *n* = 46, *p* = .361) or the number of BEs (ρ = .07, *n* = 45, *p* = .659).

**Additional file 8**

We further examined the inverse correlations between reaction RT bias variability and dwell time bias variability with binge eating frequency in the group with BED by deriving additional trial-level bias scores (TL-BS). For RT, TL-BS were calculated by pairing each incongruent trial with the next temporally contiguous congruent trial (and vice versa), provided the two occurred within five intervening trials. Within each pair, TL-BS was defined as

$$TL-BS={RT}_{congruent}-{RT}_{incongruent}$$

such that positive values indicate an attentional bias toward the affective stimulus and negative values indicate a bias away from it.

Reduced TL-BS variability may reflect (a) smaller bias excursions—i.e., lower amplitude fluctuations toward or away from affective stimuli—or (b) fewer directional shifts, implying a more stable (and potentially less flexible) attentional pattern. To determine which component underlies the relationship with binge eating episode frequency, we computed the following metrics (Zvielli et al, 2015)^[[1]](#footnote-1)^:

**Mean TL-BS positive**: the mean of TL-BS values > 0, indexing the typical magnitude of bias toward affective stimuli.

**Mean TL-BS negative:** the mean of TL-BS values < 0, indexing the typical magnitude of bias away from affective stimuli.

Additionally, we computed the following metrics to investigate the frequency of attention shifts between trials:

**Sign-change count**: the number of successive TL-BS in which the sign differed from that of the preceding score.

**Sign-change proportion**: the ratio of sign-change count to the number of valid TL-BS trials.

Dwell time TL‐BS were defined for each critical trial as the difference between dwell time on the affective stimulus and dwell time on the neutral stimulus. We then derived the same four summary metrics – mean TL‐BS positive, mean TL-BS negative, sign‐change count, and sign‐change proportion – in direct analogy to the RT parameters.

**Table AF 8**

*Descriptive parameters of trial‐level bias scores (TL-BS) and their Spearman correlations with binge eating episode frequency in individuals with binge eating disorder*

|  |  |  |  | Correlation with number of binge episodes | |
| --- | --- | --- | --- | --- | --- |
|  | ***n*** | ***M [SD]*** |  | **ρ** | ***p*** |
| Reaction time bias variability | 71 | 96.37 [7.19] |  | **-.33** | .005 |
| Mean TL-BS positive |  | 98.85 [7.12] |  | **-.37** | .001 |
| Mean TL-BS negative |  | -89.57 [7.15] |  | **.24** | .048 |
| Sign-change count |  | 23.06 [0.53] |  | .06 | .638 |
| Sign-change proportion |  | .30 [.01] |  | .06 | .639 |
| Dwell time bias variability | 65 | 549.99 [20.50] |  | **-.26** | .034 |
| Mean TL-BS positive |  | 458.09 [20.21] |  | .21 | .125 |
| Mean TL-BS negative |  | -386.16 [16.67] |  | .21 | .100 |
| Sign-change count |  | 28.63 [1.32} |  | **-.27** | .028 |
| Sign-change proportion |  | .43 [.01] |  | -.09 | .493 |

**Additional file 9**

*Statistics for 2 (training condition) x 2 (time) analysis for those with attentional bias at baseline*

| npar | *n* | Effect | ATS | df | Sig. [*p*]. |  |
| --- | --- | --- | --- | --- | --- | --- |
| Total dwell time | 46 | Time | 7.72 | 1 | **.005** |  |
|  |  | Condition | 9.25 | 1 | .002 |  |
|  |  | Time* Condition |  | 1 |  |  |
| Reaction time | 61 | Time | 9.27 | 1 | .002 |  |
|  |  | Condition | 15.86 | 1 | <.001 |  |
|  |  | Time* Condition |  | 1 |  |  |
| Direction bias | 30 | Time | 7.93 | 1 | **.005** |  |
|  |  | Group | 0.87 | 1 | .350 |  |
|  |  | Time*Group | 2.47 | 1 | .694 |  |
| Initial Fixation Duration Bias | 36 | Time | 9.32 | 1 | **.002** |  |
|  |  | Group | 0.01 | 1 | .933 |  |
|  |  | Time*Group | 0.21 | 1 | .650 |  |
| Dwell time bias | 32 | Time | 13.50 | 1 | **<.001** |  |
|  |  | Group | 2.75 | 1 | .097 |  |
|  |  | Time*Group | 2.22 | 1 | .136 |  |
| Reaction time bias | 42 | Time | 11.14 | 1 | **<.001** |  |
|  |  | Group | 0.30 | 1 | .585 |  |
|  |  | Time*Group | <0.01 | 1 | .977 |  |

1. Zvielli, A., Bernstein, A., & Koster, E. H. (2015). Temporal dynamics of attentional bias. *Clinical Psychological Science*, *3*(5), 772-788. [↑](#footnote-ref-1)
